# How Chinese Employees’ Voice Behavior Is Motivated: The Role of Perceived Overqualification

**DOI:** 10.3389/fpsyg.2022.736043

**Published:** 2022-02-17

**Authors:** Xiaoyu Wu, Fang Ma

**Affiliations:** Department of Business Administration, Business School, Beijing Technology and Business University, Beijing, China

**Keywords:** role breadth self-efficacy, perceived overqualification, leader-member exchange, voice behavior, the conservation of resources theory (COR)

## Abstract

Drawing on the conservation of resources theory (COR), we examined the effect of leader-member exchange (LMX) on the voice behavior *via* role breadth self-efficacy, and how the perceived overqualification moderates the relationship between LMX and voice behavior. We tested the theoretical model with data gathered from 407 individuals in China. The results revealed that LMX had an indirect effect on voice behavior through role breadth self-efficacy, and perceived overqualification moderated the positive association between LMX and role breadth self-efficacy. In addition, the mediating effect of LMX on voice behavior through role breadth self-efficacy was stronger when the level of perceived overqualification was low and weaker when it was high. The findings have theoretical and practical implications for increasing employees’ voice behavior in organizations.

## Introduction

In VUCA (volatility, uncertainty, complexity, and ambiguity) era, the fierce external competition environment makes it impossible to face all issues only by the wisdom of leaders. In order to enhance the competitive advantage, organizations need to rely on the positive work attitude and behaviors of employees. And the employee’s voice behavior, as proactive behavior, can promote the development and innovation of organization (Sun and Pan, 2017). But voice behavior is challenging and transformative in nature, therefore, voice behavior is often accompanied by conflict or risk, which made employees more cautious about voice. Therefore, employees often choose to “know but keep silent,” and tend to wisely protect themselves. The phenomena are particularly obvious in China’s organization, where harmony is valued and with higher power distance.

Voice behavior is an interaction between leaders, organizations and employees (Van Dyne and LePine, 1998), and the quality of the relationship determines the results of voice behavior. Guanxi orientation, originated from Confucianism, is an important theme in Chinese organization and society (Tian and Zhang, 2019; Wu, 2020). Intimacy is an important basis for interpersonal interaction and behavior. Leaders often classify their employees as “insiders” or “outsiders” due to limited resources, time pressure and other restrictive factors when establishing relations with employees, which is leader-member exchange (namely LMX) (Graen et al., 1972). Prior studies on LMX and voice behavior mainly focused on the western cultural background. Although Chinese scholars started to explore the mechanism between LMX and voice behavior recently, the conclusion focuses on two perspectives. One is that when leaders provide more resources to support the employees who is “insiders,” these employees will give back and maintain this exchange relationship to voice behavior (Qi and Yang, 2018). The other is that employees dare to make suggestions just because they have a good relationship with their leaders. However, voice behavior is a challenge to the current situation, which is likely to annoy leaders and cause interpersonal conflict (Van Dyne and LePine, 1998). Neither perspective provided an appropriate explanation. According to the conservation of resources theory (COR), employees try to protect and build valuable resources, but whether the resources can be effectively used in their work depends on the beneficial resource sharing provided by leaders and accumulated to form stock, not on short-term exchange behavior (Jiang et al., 2019). Therefore, based on COR, this study takes LMX as the resource factor acquired by employees to explore the effect of LMX on employee voice behavior in Chinese context.

Parker et al. (2010) proposed that LMX is the distal antecedent of employee behavior, which acts through employees’ psychological state. Previous studies showed that role breadth self-efficacy, a valuable psychological resource of employees, can predict proactive behavior, reflecting the confidence on whether employee complete a wider range of tasks. High-quality LMX affects employees’ judgment of their ability, and then increases employees’ own resource level, thus influencing their voice behavior. Therefore, in the framework of COR, this study introduced role breadth self-efficacy, an individual characteristic variable closely related to personal resources, to explore whether it plays a mediating role between LMX and voice behavior.

**FIGURE 1:**
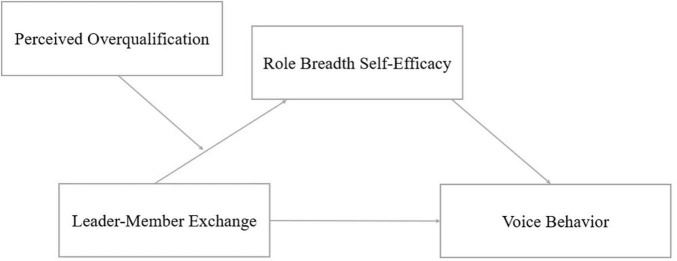
Theoretical model.

According to prior research, it referred that the relationship between leaders and employees affected the judgment on employees’ ability, but the degree of influence may be affected by the characteristics of individual differences (Chen, 2019). Due to the pandemic of COVID-19, there is an imbalance between the demand for high-quality jobs and the supply of jobs. Employees have to choose jobs that are lower than their qualifications, which leads to the feeling that they are big fish in a small pond, namely perceived overqualification. When employees perceived a high level of overqualification, their talent cannot be fully displayed, which is not only a waste of their resources, but also hinders the process of accumulating more resources by making full use of their qualifications. Therefore, employees will feel the loss of their resources. Therefore, even in the better LMX, overqualified employees tend to show a state of self-protection in order to avoid further loss of resources due to the lack of growth platform, thus inhibiting role breadth self-efficacy. Combined with the view of COR that the interaction effect of resources between individuals and environment will affect individuals (Cao and Qu, 2014), this study intends to analyze the moderating effect of perceived overqualification on LMX and role breadth self-efficacy. The graphical representation of the proposed model is provided in Figure 1.

This study makes several contributions to the literature. Firstly, prior studies on the mechanism of voice behavior are mostly based on social exchange theory. Detert and Edmondson (2011) put forward that many people are still unwilling to repay their organization through voice behavior even under an autonomous and supportive environment. Therefore, using social exchange theory to explain the mechanism of voice behavior is not comprehensive. Based on COR, Jiang et al. (2019) proposed that high-quality LMX is matched by employees’ positive work attitude, outstanding work performance and even their demographic characteristics similar to their leaders, and the resource support brought by leaders can promote the appreciation of employees’ own resources. From the perspective of COR, this study takes LMX as resources that employees get from their leaders, and explores the effect of LMX on voice behavior based on the context of Chinese organizations, which enriches the theoretical system of localized LMX and provides a basis for subsequent empirical studies.

Secondly, under the framework of COR, this study constructs a theoretical model of LMX by taking role breadth self-efficacy as mediating role, and uses empirical method to analyze the mechanism of LMX on employee voice behavior, so as to reveal the “black box” of LMX on employee voice behavior. At the same time, the role of individual psychological resources in voice behavior was analyzed, which further enriched COR.

Thirdly, this study introduces perceived overqualification into the framework of LMX and test its moderating effect, which further improved and developed the contingency perspective of LMX related research. Empirical studies have found that with the expansion of higher education, more and more talents enter the labor market, which makes more difficult for employees to find jobs that match their qualifications. A survey shows that 84% of Chinese employees feel overqualified, ranking first in the world (Lin et al., 2017). In this context, it is of great significance to take perceived overqualification as the moderating role to explore the influence of LMX, which will help to better understand the importance of person-job fit on recruitment.

## Theory and Hypothese

### Leader-Member Exchange and Voice Behavior

Leader-Member Exchange refers to that leaders classify subordinates as “insiders” and “outsiders” because of limited resources, time pressure and other restrictive factors (Graen et al., 1972), which reflects the quality of the relationship between leaders and subordinates. When the relationship between leaders and subordinates is of high-quality, leaders will regard employee as “insiders” and maintain a high level of mutual trust, support and respect between them, and employees are more likely to receive formal or informal supports or rewards. On the contrary, when the relationship quality is low, employees will be regarded as “outsiders,” who can only get authorization within the scope of work and maintain a lower level of interaction, trust or support with the leader. High-quality LMX are mature partnerships characterized by mutual trust and respect. Previous studies have shown that a good relationship between leaders and employees can effectively stimulate employees’ proactive behavior. Voice behavior is one of the proactive behaviors, and leaders are often the target of employee voice. Their attitude and behavior will affect employee’s willingness to voice, so the relationship with leaders will also affect employee voice behavior (Duan et al., 2017).

Leaders usually maintain most scarce resources and information in organizations. Employees who have a high-quality relationship with their leader will gain more trust, care and resources, and have better psychological and work feelings (Liang et al., 2019). Based on COR, high-quality relationship between leaders and employees is an important organizational and psychological resource for employees, which can improve employees’ judgment on the success rate of voice behavior and reduce their perception of the risk of voice, thus affecting voice behavior (Liang et al., 2019). High-quality LMX will make employees feel respect and trust by their leaders, which will reduce employees’ risk perception of voice, increase their psychological security (Ashford et al., 1998; Morrison, 2011), and thus have a higher possibility to conduct voice behavior. In addition, when the voice behavior of the close employees achieves good results, the leader will think it is the embodiment of the employee’s ability, further enhance trust and emotion, and give them more rewards and resources, thus increasing the resource stock of the employees (Liang et al., 2019). When voice behavior fails to achieve the desired effect, the leader will also try to understand them, so as to avoid the depletion of employees’ resources (Wang et al., 2010). On the contrary, employees under lower LMX have little contact with their leaders, receive few resources and limited information (Mowbray et al., 2015). The voice behavior from subordinates may be mistaken as a challenge to the authority of the leader, resulting in the depletion of staff resources (Liang et al., 2019). Therefore, employees who have a good relationship with leaders will actively voice to obtain more resources, otherwise, they will keep silence or reduce voice behavior to avoid resource depletion. Therefore, the hypothesis is proposed as follows:

H1: LMX is positively associated with employees’ voice behavior.

### The Mediating Role of Role Breadth Self-Efficacy

Role breadth self-efficacy refers to employees’ perceived ability to perform a wider range of tasks and assume a wider range of roles beyond the prescribed technical requirements. It is a new concept based on self-efficacy (Parker, 1998), and is a positive psychological resource (Zhu et al., 2020). Compared with self-efficacy, role breadth self-efficacy pays more attention to extra-role behaviors (Galperin, 2012). Therefore, the relationship between role breadth self-efficacy and voice behavior is closer. Previous researches showed that trust and positive individual behavior of superiors could improve employees’ role breadth self-efficacy (Axtell and Parker, 2003; Parker et al., 2006; Huang and Peng, 2015), and the strengthening of role breadth self-efficacy can promote employees’ proactive behavior (Zhang et al., 2016).

Bandura (1977) proposed that the formation of self-efficacy comes from the following four factors, performance accomplishments; vicarious experience, that is, observing and imitating the behavior of others; verbal persuasion, that is, encouragement and positive evaluation of others; and physiological states. Role breadth self-efficacy, as an extension and development of self-efficacy, can also be improved based on the mechanism mentioned above (Wang, 2017). Firstly, Huang and Peng (2015) believed that high-quality interactive relationship between leaders and employees is an important way for employees to obtain resources. The resources and support obtained constitute the information source for employees to evaluate their ability, thus shaping employees’ efficacy belief (Gist and Mitchell, 1992). Secondly, Bandura (1977) pointed out that encouragement and positive evaluation from leaders could enhance employees’ judgment of their own abilities. In high-quality LMX, leaders tend to give more resources for employees, showing the trust and appreciation to them. At the same time, the employees also fully trust and understand their leaders. These emotional interactions will enhance employees’ judgment of their own abilities, and thus improve their role breadth self-efficacy. Thirdly, Xia et al. (2020) found that high-quality LMX significantly improved employees’ security and reduce their psychological burden. This makes employees present a positive psychological state in the work process of work, and then improves role breadth self-efficacy. Therefore, the hypothesis is proposed as follows:

H2: LMX is positively associated with employees’ role breadth self-efficacy.

According to COR, in order to realize the appreciation of existing resources, employees will take actions to seek and save resources (Qu et al., 2014). Therefore, role breadth self-efficacy, as a positive psychological resource, reflects the “can do” motivation (Xu et al., 2018), and affects employees’ behavior (Gao and Yuan, 2019). Employees with higher role breadth self-efficacy are more confident on completing a series of extra-role tasks and engaging in activities, and able to propose corresponding solutions to problems in organization (Liao and Liang, 2015). Given the impact of voice behavior on the operation and innovation of organization, employees who perform voice behavior can obtain more recognition and other resources, which can motivate employees to obtain these resources through voice (Qu et al., 2014). Therefore, employees with high role breadth self-efficacy may regard voice behavior as an opportunity to acquire resources and thus voice more. Therefore, role breadth self-efficacy has a positive effect on the formation of employee voice behavior.

From the above analysis, it can be inferred that LMX is likely to influence voice behavior through positive effect on employees’ role breadth self-efficacy. Parker et al. (2010) pointed out that the relationship between leader and employee is a distal factor affecting employee behavior, and its effect is played through the proximal psychological variable, that is, role breadth self-efficacy, is the mediating mechanism of LMX affecting employee behavior. Thus the hypothesis is proposed as follows:

H3: Role breadth self-efficacy mediates the relationship between LMX and employees’ voice behavior.

### The Moderating Role of Perceived Overqualification

Overqualification arises from underemployment (McKee-Ryan and Harvey, 2011) or overeducation (Burris, 1983), including both objective and subjective levels (Yang and Zhou, 2013). Objective levels of overqualification refers to the educational level, knowledge, experience, skills and ability of individuals higher than the actual job requirements, which is mainly measured by job analysis and comparison (Erdogan and Bauer, 2009; Maltarich et al., 2011). The subjective perception, which is perceived overqualification, refers to “employee perceptions of surplus education, experience, and KSAs (knowledge, skills, and abilities)” (Maynard et al., 2006). Most researches focus on perceived overqualification, because of objective overqualification does not explain how similarly qualified people in similar jobs experience varying degrees of feeling overqualified (Erdogan et al., 2011). Previous studies on perceived overqualification tend to take one of two perspectives, namely, the frustration caused by underutilization of qualification and capability-based perspective (Wu et al., 2017). According to the first view, perceived overqualification would lead to negative work attitudes and behaviors among employees because of the underutilization and frustration (Maynard et al., 2006; Maynard and Parfyonova, 2013; Alfes et al., 2016; Erdogan and Bauer, 2021). However, from the capability-based perspective, it treated overqualification as the availability of, or self-assessments relating to, surplus skills and qualifications. This perspective focuses on the high level of ability of employees to respond to job demands and perform well at work (Erdogan and Bauer, 2021). Therefore, the effects of perceived overqualification on employees’ attitude, psychological state, behaviors are also different depending on the perspective adopted. For example, Chu et al. (2021) based on social cognitive theory, considered that employees who perceived overqualified turn their attention to excess ability, and their remaining qualifications could be used to perform extended roles, which is crucial to the formation of role breadth self-efficacy. Drawing on social cognitive theory of self–regulation, Zhang et al. (2016) found that perceived overqualification made employees feel task was simpler when analyzing task requirements, and feel less constraint in the process of task execution, as well as they are more likely to attribute achievement of goals to their own abilities, thus prompts the role breadth self-efficacy. On the contrary, Zhao et al. (2019) put forward that the overqualified employees think that the current job causes a waste of resources such as knowledge, skills and time, and hinders them from developing new skills, thus resulting in psychological frustration and emotional exhaustion. Li et al. (2020) regarded overqualification as underutilization of skills. Based on self-representation theory, they found that perceived overqualification would lead to negative emotions such as frustration, anger and hostility, thus reduce organizational self-esteem and bring psychological pressure to employees. COR holds that the sense of resource loss of overqualified employees will lead to negative physical and mental outcomes, work attitudes and behaviors (Wassermann and Hoppe, 2019; Li et al., 2021). Therefore, grounding on the COR, this study takes the view that the frustration caused by underutilization of qualification to explore perceived overqualification, specifically, in the employment situation where the qualifications are not fully utilized. Overqualified employees will feel loss of resources. It then causes negative work attitude and emotions, which will have a negative impact on the role breadth self-efficacy.

In addition, perceived overqualification is often regarded as an individual factor that directly affects employees’ work mentality, work behaviors and work performance. However, some scholars also discussed the influence of perceived overqualification from the perspective of moderating effect. For instance, based on the self-determination theory, studies verified perceived qualification had a negative moderating effect on the relationship between career growth and the satisfaction of basic psychological needs of employees (Zhang and Li, 2020). However, the research perspective of perceived overqualification as a negative situational factor has not received widespread attention. Prior studies mainly discussed the separate effects of different LMX mechanisms, but lack of studies on the mechanism of LMX under different individual characteristics. Therefore, this study introduced perceived overqualification to explore the moderating effect of perceived overqualification on the relationship between LMX and role breadth self-efficacy.

Conservation of resources theory believes that the employees will invest a lot of time, energy and social relations to improve their self-value, and expect the spiral of resource appreciation rather than loss (Cao and Qu, 2014). Perceived overqualification means the imbalance between resource input and reward, and leads to more negative emotional reactions (Zhao et al., 2019), which is not conducive to the formation of role breadth self-efficacy (Bandura, 1977). In addition, the speed of resource loss is faster than value appreciation (Jiang et al., 2019). A lack of leadership and organizational identification, employees with higher level of perceived overqualification have the feeling of lower insider identity (Zhao et al., 2018), and have difficulties in gaining resources (Jiang et al., 2019). Therefore, they are more likely to fall into the spiral of loss, and more sensitive to the loss of resources, thus will put the protection of existing resources as the first principle, and less likely to take on the idea of undertaking a wider range of tasks. Therefore, even under LMX, employees with higher level of perceived overqualification tend to act conventionally and show avoidance state of self-protection, thus inhibiting role breadth self-efficacy. Based on this, the following hypothesis is proposed:

H4: Perceived overqualification moderates the relationship between LMX and role breadth self-efficacy, such that the positive effect is stronger when the level of perceived overqualification is low than when it is high.

H1 to H4 present the relationships constituting the overall moderated mediation model. This study further examined whether perceived overqualification not only moderates the relationship between LMX and role breadth self-efficacy but also moderates the indirect effect of LMX on employees’ voice behavior through role breadth self-efficacy. Finally, we proposed the following hypothesis.

H5: Perceived overqualification moderates the relationship between LMX and voice behavior through role breadth self-efficacy, such that the indirect effect is stronger when the level of perceived overqualification is low than when it is high.

## Materials and Methods

### Participants and Procedure

A questionnaire survey was designed to collect research data from November 2020 to January 2021, targeting at manufacturing, IT operation, and catering service in different regions of China. We conducted the survey through the human resource departments of each firm. We designed two formal surveys for each firm with an interval of 2 months to avoid common method variance. The first survey was distributed to 422 employees to measure demographics, LMX, perceived overqualification. Two months later, the second survey was conducted to the same respondents to measure role breadth self-efficacy and voice behavior. Four hundred and seven matched data were found available for final analysis, and the effective recovery was 96.89%.

Of the respondents, 50.61% were male, 49.39% were female. According to the education background, the distribution of senior high school and below accounts for 4.67%, junior college for 11.55%, bachelor’s for 70.52%, master for 12.29% and doctor accounts for 0.98%. The 164 (40.29%) respondents were of age of 26–30 years, 134 (32.92%) were of age of 31–40 years, 93 (22.85%) were of age of 18–25, 13 (3.19%) were of age of 41–50, only 3 (0.74%) were of age of 51–60 and no one has more than 60 years of age. In terms of experience, 147 (36.12%) had more than 5 years experiences, and 111 (27.27%) had 3–5 years experiences. 149 (36.61%) had less than 3 years experiences.

Guarantees of confidentiality and anonymity were provided to respondents to reduce respondent anxiety or answers based on their actual feelings.

### Measures

The questionnaire was first designed in English and then translated to Chinese by two bilingual academic researchers, using a back-to-back translation method (Brislin, 1986). A panel discussion was conducted to enhance the validity of this research. Participants of the panel discussion included university scholars, employees and managers. A pilot test was undertaken with 50 respondents. The questionnaire was finalized, and four sections were included.

#### Leader-Member Exchange

The LMX items were adopted from Graen and Uhl-Bien (1995). The seven items were rated on a five-point Likert scale (ranging from 1 = strongly disagree to 5 = strongly agree). A sample item is “My leader pays attention to the needs of employees” (Cronbach’s α = 0.87; see Table 2).

#### Voice Behavior

The voice behavior items were adopted from Liang et al. (2012). The ten items were rated on a five-point Likert scale (ranging from 1 = strongly disagree to 5 = strongly agree). In this study, eight items were reserved according to factor load. A sample item is “I will actively make developmental suggestions on issues that have an impact on the organization or department” (Cronbach’s α = 0.81; see Table 2).

#### Role Breadth Self-Efficacy

The role breadth self-efficacy items were adopted from Parker et al. (2006). The seven items were rated on a five-point Likert scale (ranging from 1 = strongly disagree to 5 = strongly agree). A sample item is “I can design new procedures in my field” (Cronbach’s α = 0.82; see Table 2).

#### Perceived Overqualification

The perceived overqualification items were adopted from Maynard et al. (2006). The nine items were rated on a five-point Likert scale (ranging from 1 = strongly disagree to 5 = strongly agree). In this study, six items were reserved according to factor load. A sample item is “Even though I do not have previous work experience, I can successfully complete my current job” (Cronbach’s α = 0.77; see Table 2).

#### Control Variables

Previous empirical studies have confirmed that gender and education is positively related to employees’ voice behavior (Zhou and Liao, 2013). Following prior suggestions to use control variables, we controlled for employees’ gender and education to better estimate the effects of LMX on voice behavior.

## Results

### Confirmatory Factor Analysis

A Confirmatory Factor Analysis of the above four measures was conducted to analyze discriminant validity using AMOS 23.0 with maximum likelihood estimation procedures. As shown in Table 1, we found good support for the four-factor solution (LMX, voice behavior, role breadth self-efficacy, and perceived overqualification), which showed an adequate fit to the data: χ^2^ = 686.63, degrees of freedom (*df*) = 344, comparative fit index (CFI) = 0.92, incremental fit index (IFI) = 0.92, Tucker-Lewis index (TLI) = 0.91, and root mean square error of approximation (RMSEA) = 0.050.

**TABLE 1:** Confirmatory factor analysis.

Model	Factor	χ^2^	*df*	χ^2^/*df*	CFI	TLI	IFI	RMSEA
Model 1	Four-factor model (POQ, RBSE, VB, LMX)	686.83	344	1.997	0.92	0.91	0.92	0.050
Model 2	Three-factor model (POQ + RBSE, VB, LMX)	1186.48	347	3.419	0.79	0.77	0.79	0.077
Model 3	Two-factor model (POQ + RBSE, VB + LMX)	1614.77	349	4.627	0.69	0.66	0.69	0.095
Model 4	Two-factor model (POQ + LMX, VB + RBSE)	1224.66	349	3.509	0.78	0.76	0.78	0.079
Model 5	One-factor model (POQ + RBSE + VB + LMX)	1775.32	350	5.072	0.65	0.62	0.65	0.100

*df, degrees of freedom; CFI, comparative fit index; TLI, Tucker-Lewis index; IFI, incremental fit index; RMSEA, root mean square error of approximation; POQ, perceived overqualification; RBSE, role breadth self-efficacy; VB, voice behavior; LMX, leader-member exchange.*

Next, Harman’s single factor test was conducted to test the common method deviation of the variables. The one-factor model is very unsatisfactory. All items were included in the factor analysis, and the first principal component was obtained without rotation, which explained the variation of the total variance of 25.495%, which was lower than 40%, indicating that the deviation of the common method was not an issue in the current study.

### Descriptive Statistics and Correlations

The means, standard deviations, and correlations for each variable are provided in Table 2. The results showed that LMX was positively and significantly related to role breadth self-efficacy (*r* = 0.46, *p* < 0.01) and voice behavior (*r* = 0.54, *p* < 0.01). As expected, LMX was negatively and significantly related to perceived overqualification (*r* = −0.21, *p* < 0.01). Thus, the correlation results were in line with theoretical expectations and provided a basis support for further analysis.

**TABLE 2:** Results of the descriptive statistical analysis.

	M	SD	1	2	3	4	5	6
1. Gender	1.49	0.50						
2. Education	2.93	0.68	0.00					
3. LMX	3.64	0.68	0.01	0.13*	(0.87)			
4. RBSE	3.75	0.61	−0.11*	0.29**	0.46**	(0.82)		
5. VB	3.80	0.57	−0.17**	0.12*	0.54**	0.71**	(0.81)	
6. POQ	2.86	0.74	0.02	–0.02	−0.21**	−0.17**	−0.18**	(0.77)

*N = 407; Cronbach’s alpha reliability coefficients are displayed on the diagonal.*

*LMX, leader-member exchange; RBSE, role breadth self-efficacy; VB, voice behavior; POQ, perceived overqualification.*

*P < 0.05; **P < 0.01.

*Male = 1; Female = 2.*

*High school and below = 1; College degree = 2; Bachelor’s degree = 3; Master’s degree = 4; and Doctor’s degree and over = 5.*

Since role breadth self-efficacy is highly correlated with voice behavior (*r* = 0.71, *p* < 0.01), confirmatory factor analysis is further conducted for both, and the results are shown in Table 3. For items of role breadth self-efficacy, confirmatory factor analysis showed that the fit indexes of the scale were χ^2^/*df* = 2.402, CFI = 0.975, TLI = 0.962, IFI = 0.975, and RMSEA = 0.059, indicating that the scale had good structural validity. At the same time, CR was 0.97, AVE was 0.82, which were all up to standard. As for voice behavior, confirmatory factor analysis showed that the fit indexes of the scale were χ^2^/*df* = 2.157, CFI = 0.977, TLI = 0.964, IFI = 0.977, and RMSEA = 0.053, indicating that the scale had good structural validity. As well as CR and AVE were meet statistical requirements (CR = 0.99, AVE = 0.95).

**TABLE 3:** Confirmatory factor analysis of RBSE and voice behavior.

Variable	χ^2^	*df*	χ^2^/*df*	CFI	TLI	IFI	RMSEA	CR	AVE
RBSE	33.621	14	2.402	0.975	0.962	0.975	0.059	0.97	0.82
VB	38.831	18	2.157	0.977	0.964	0.977	0.053	0.99	0.95

*N = 407. RBSE, role breadth self-efficacy; VB, voice behavior.*

### Hypothesis Testing

Firstly, we tested the indirect effects. As shown in Table 4, the results from the regression analysis of the mediation model indicated that LMX was positively and significantly associated with voice behavior (model 4; β = 0.45, *p* < 0.001) and role breadth self-efficacy (model 2; β = 0.39, *p* < 0.001). Role breadth self-efficacy was positively and significantly associated with voice behavior (model 5; β = 0.55, *p* < 0.001). Thus, Hypothesis 1 and 2 were supported. After controlling for role breadth self-efficacy, the relationship between LMX and voice behavior is significantly positive, but the level of effect is significantly decreased (model 5; β = 0.24, *p* < 0.001). The result showed that the indirect effect of LMX on voice behavior was significant. Thus, Hypothesis 3 was supported.

**TABLE 4:** Mediating effect of role breadth self-efficacy.

Variables	RBSE		VB		
	Model 1	Model 2	Model 3	Model 4	Model 5
Gender	−0.14*	−0.15**	−0.19***	−0.20***	−0.12**
Education	0.27***	0.22***	0.10*	0.04	−0.08**
LMX		0.39***		0.45***	0.24***
RBSE					0.55***
F	22.39***	52.57***	8.98***	65.52***	140.38***
*R* ^2^	0.10	0.28	0.04	0.33	0.58
Δ*R*^2^	0.10	0.18	0.04	0.29	0.26

*N = 407.*

*LMX, leader-member exchange; RBSE, role breadth self-efficacy; VB, voice behavior.*

*P < 0.05; **P < 0.01; and ***P < 0.001.

**FIGURE 2:**
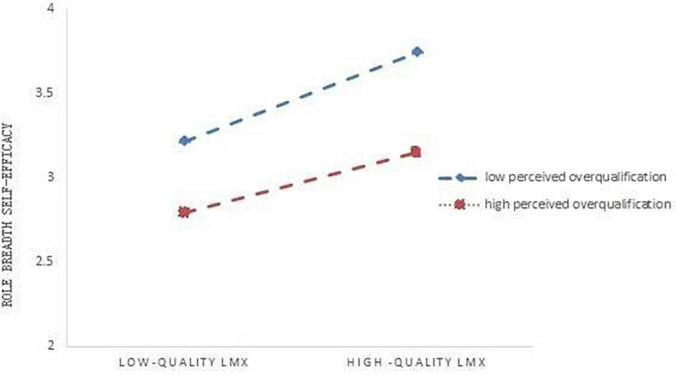
Moderating effect of perceived overqualification on the relationship between LMX and role breadth self-efficacy.

Secondly, we tested the moderation model by using regression analysis. As shown in Table 5, the interaction term of LMX and perceived overqualification was statistically significant (β = −0.12, *p* < 0.05). Figure 2 further revealed that the positive relationship between LMX and role breadth self-efficacy was stronger for people with lower levels of perceived overqualification. Thus, Hypothesis 4 was supported.

**TABLE 5:** Moderating effect of perceived overqualification.

Variables	RBSE			
	Model 1	Model 2	Model 3	Model 4
Gender	−0.14*	−0.15**	−0.14**	−0.14**
Education	0.27***	0.22***	0.22***	0.22***
LMX		0.39***	0.37***	0.39***
POQ			–0.06	–0.06
LMX × POQ				−0.12*
F	22.39***	52.57***	40.40***	33.94***
*R* ^2^	0.10	0.28	0.29	0.30
Δ*R*^2^	0.10	0.18	0.01	0.01

*N = 407.*

*LMX, leader-member exchange; RBSE, role breadth self-efficacy; POQ, perceived overqualification.*

*P < 0.05; **P < 0.01; and ***P < 0.001.

Thirdly, to test the conditional indirect effect, we assessed the moderated mediation model using 5,000 bootstrap estimates based on 95% bias-corrected CIs (PROCESS, model 7). As indicated in Table 6, when the level of perceived overqualification was low, the relationship between LMX and voice behavior through role breadth self-efficacy was significant and positive [indirect effect = 0.2634, 95% CI (0.1915, 0.3514), excluding 0]. Correspondingly, when the level of perceived overqualification was high, the relationship between LMX and voice behavior through role breadth self-efficacy was still positive, but the relationship was weaker [indirect effect = 0.1644, 95% CI (0.0986, 0.2457), excluding 0]. Furthermore, the difference between the two levels was significant, with 95% CI (−0.1418, −0.0023), excluding 0. Thus, Hypothesis 5 was supported.

**TABLE 6:** Results of the moderated mediation effect between leader-member exchange (LMX) and voice behavior.

Moderator	Level	Effect	Boot SE	Boot *p*	95% CI
Perceived	Low (−1 SD)	0.2634	0.0405	0.000	(0.1915, 0.3514)
overqualification	High (+ 1 SD)	0.1644	0.0370	0.000	(0.0986, 0.2457)

*N = 407.*

*Bootstrapping repetitions, N = 5,000.*

*CI, confidence interval.*

## Discussion

Grounding on the COR, our study developed a theoretical model of LMX and voice behavior. We took LMX as the antecedent of voice behavior and introduced role breadth self-efficacy to explain the mechanism through which LMX affected voice behavior, from the new perspective of resources. In addition, our study added perceived overqualification as a boundary condition. From a sample of 407 participants, the results revealed that LMX was positively related to voice behavior through role breadth self-efficacy, and that perceived overqualification moderated the positive relationship between LMX and role breadth self-efficacy, such that the relationship was weaker for individuals with higher levels of perceived overqualification. We also found that the indirect effect of LMX on voice behavior was moderated by perceived overqualification.

### Theoretical Implications

Our study makes several contributions to the literature. First, voice behavior is an extra-role interpersonal communication behavior in which individuals aim to improve their work or organizational status, are reform-oriented, and have constructive opinions (Van Dyne and LePine, 1998). Based on COR, this study found that LMX can promote the generation of voice behavior from the perspective of resources, thus enriching the research perspective of voice behavior mechanism.

Second, from the perspective of COR, role breadth self-efficacy is regarded as a positive psychological resource, and the motivation behind employee with role breadth self-efficacy participated in voice is analyzed, that is, voice behavior is an important way for employees to obtain new resources. This shows that employee’s voice behavior not only brings benefits to the organization, but also brings more resources to themselves. This finding explains the relationship between role breadth self-efficacy and voice behavior from a new perspective, and clearly presents the process of LMX effect on voice behavior.

Finally, perceived overqualification was introduced into the pathway of “LMX-Role Breadth Self-Efficacy-Voice Behavior” to verify the moderating effect of individual trait differences. It is found that different individuals respond differently to LMX. Employees who perceived overqualified underutilized their skills in the current job, and will have a stronger negative impact on role breadth self-efficacy. This finding also supports the view that perceived overqualification may have a more serious impact on individuals themselves (Smith and Frank, 2005). In other words, perceived overqualification means that the abilities of employees are underutilized and it is difficult to give full play to their qualifications in work, which is a gap between the actual state and the ideal state (Ding et al., 2019). This psychological gap will directly lead to negative attitudes and emotions toward work (Johnson and Johnson, 2000; Kristof-Brown et al., 2005; Gabriel et al., 2014), which in turn reduces role breadth self-efficacy of employees (Bandura, 1977). Zhang and Li (2020) considered perceived overqualification as underutilization and frustration, which may inhibit the satisfaction of psychological state brought by career growth. Yang and Li (2021) found that perceived overqualification led to frustration and positively affects negative emotions. In a specific cultural context, perceived overqualification has a negative impact on positive self-concept. The essence of these conclusions is to regard overqualification as a waste of resources and underutilized capacity in the organization. The findings of this study are crucial for understanding how to help employees who perceived overqualified adjust and utilize resources.

In addition, this study further verified perceived overqualification moderates the indirect effect of LMX on voice behavior through role breadth self-efficacy, and found the boundary conditions of the LMX, which has a good enlightenment and significance for further exploring and deepening the research on LMX.

### Practical Implications

This study provides guidance to organizational management and human resource practices. First, establishing a high-quality LMX within the organization. In order to stimulate voice behavior, leaders should build a “circle” culture and avoid treating employees alike, which is quite different from the traditional perception. This puts forward higher requirements for leaders, namely, the lower quality LMX will frustrate the initiative of voice due to lack of resources. Therefore, leaders should develop LMX according to the actual situation of organization.

Second, managers can adopt measures to improve employees’ role breadth self-efficacy. Specifically, managers should praise employees more, encourage them, give positive comments on their suggestions, and view employees’ initiative with a positive attitude. This could not only improve employees’ confidence on engaging in proactive behavior, and then enhance their role breadth self-efficacy (Huang and Peng, 2015), but also show that the organization holds a positive and welcoming attitude toward employees’ voice behavior, so as to promote more employees to implement voice behavior (Morrison and Milliken, 2000).

Finally, this study found that perceived overqualification can reduce the positive relationship between LMX and role breadth self-efficacy, which suggests that managers should pay attention to employees’ overqualification. They need to be aware of the risks associated with recruiting highly qualified people. In the hiring process, try to hire the people who match the job. Furthermore, for the employees with high level of perceived overqualification, measures such as regular attention to their psychological state and training need to be taken to correctly guide the self-evaluation of the employees and timely intervene the employees who are in a higher level of perceived overqualification.

### Limitations and Directions for Future Research

Our study also has some limitations that provide directions for future research. Firstly, although the self-reported responses by the participants can measure the subjective feelings of the participants more accurately, the object of employee’s voice behavior is generally superiors or colleagues, so the data obtained by this method may be deviated from the reality. Future research should adopt a method of pairing between superiors and subordinates or between colleagues to better identify the causal relationship between LMX and voice behavior.

Secondly, the selection of control variables is not comprehensive enough. Only demographic variables were considered as the control variables in this study. However, an individual’s emotions and other characteristics will affect his or her behaviors. For example, their negative emotions will lead to counterproductive behaviors (Liu et al., 2015). Therefore, future research should try to take employee emotion and other characteristics as control variables.

Finally, although this study examines the boundary conditions of LMX on voice behavior from the individual level, it ignores the influence of the level of other colleagues’ perceived overqualification. Research shows that peer-group perceptions of overqualification can affect the performance of employees (Alfes, 2013). Therefore, future research need to explore the moderating effect of perceived overqualification of colleagues on the above mechanisms.

## Conclusion

The studies we conducted revealed that LMX can increase employees’ voice behavior by increasing their role breadth self-efficacy, in addition, perceived overqualification can moderate the mediated relationship. Our research also provides the basis for practical recommendations for increasing the employee’s voice behavior.

## Data Availability Statement

The original contributions presented in the study are included in the article/supplementary material, further inquiries can be directed to the corresponding author.

## Ethics Statement

Ethical review and approval was not required for the study on human participants in accordance with the local legislation and institutional requirements. The patients/participants provided their written informed consent to participate in this study.

## Author Contributions

Both authors have contributed significantly and approved the submitted version.

## Conflict of Interest

The authors declare that the research was conducted in the absence of any commercial or financial relationships that could be construed as a potential conflict of interest.

## Publisher’s Note

All claims expressed in this article are solely those of the authors and do not necessarily represent those of their affiliated organizations, or those of the publisher, the editors and the reviewers. Any product that may be evaluated in this article, or claim that may be made by its manufacturer, is not guaranteed or endorsed by the publisher.
